# A Case of Occulsion of the Vagina

**Published:** 1881-03

**Authors:** Jno. G. Earnest

**Affiliations:** Newnan, Georgia


### A CASE OF OCCULSION OF THE VAGINA.

BY JNO. G. EARNEST, M.D., OF NEWNAN, GEORGIA.

On the 10th of December 1873, A. L. called to see me, and stated that she was thirty-four years old, had four children, had enjoyed good health until two years previous, when she "took cold" during a menstrual period. A profuse leucorrhœa set in, accompanied with fever and excessive tenderness of the lower part of the abdomen with dysurea. A few days later she injected into the vagina some fluid given her by her physician. This was followed by severe pain, lasting several hours, and an increase of temperature and also a marked decrease in the quantity of the discharge from the vagina. When she at the end of five or six weeks was able to leave her bed, she found herself "grown up." From that time she suffered inconvenience in attempting to reach up with her hands for objects higher than her head. Sexual intercourse has been impossible and menstruation painful.

The vagina was found to be occluded by what might have been mistaken for a congenital deformity but for the previous history. Neither touch or sight could detect anything like a cicatrix. The union of the surfaces extended diagonally across the vagina from a point on the left side an inch from the vulva to a point an inch and a half higher upon the opposite side. At this point an opening large enough to admit a Sims sound was found. Introducing the index finger into the rectum the neck of the uterus was felt far back pointing toward the sacrum. As it was impossible to detect any difference in the appearance of the tissue over the line of union, as the parts were necessarily put on the stretch, thereby obliterating the angle at that point, it was necessary to exercise great care in selecting the initial line of incision; and in fact, this intimate union was maintained nearly over the whole of the surfaces united, so that it was necessary to feel the way cautiously with the left index in the rectum and a sound in the bladder, until by a careful and tedious dissection, in the face of a troublesome hemorrhage, the vagina was finally restored and the uterus freed from its imprisonment. Upon opening the sac formed by the cicatrix and the upper portion of the vagina about a drachm of offensive pus escaped, having strong catamenial odor. There was erosion of the neck and a granular condition of the patulous os. The vagina was firmly packed with cotton saturated with glycerine, which stopped the hemorrhage, and was not removed until the fourth day, when it was found to have loosened. After its removal the vagina was washed out with a stream of warm water and loosely tamponed again with cotton soaked in glycerine. This was repeated daily for twenty-one days. The surfaces were at that time found to have healed, and only a slight contraction of the vagina found to exist. The cotton was discontinued and the patient ordered to continue the free injection of warm water every morning and evening and report in a few weeks.

January 24, 1874. The patient was examined and the vagina found to present a natural appearance except over an elliptical surface over half an inch wide by an inch long, stretched transversely across the vagina on the left side at the point where the slight contraction existed. Over this surface the color was pale and the tissue dense.

Removing from the locality soon after this I lost sight of the case until I met her husband, who informed me that soon after I last saw her she became pregnant and was delivered at full term and without any untoward complication, and has since enjoyed very good health.

This case is interesting on account of the intimate union over so large a surface and apparent absence of cicatricial tissue over the most of it, suggesting a possibility of the conversion of the original cicatrix that united these surfaces into tissue analogous to the surfaces it united. Having operated on several cases of occlusion of the vagina, the author has not met with any other where such intimate relations between the united surfaces existed, or where so little disposition to contraction was manifested afterward. The case seems to sustain the opinion advanced by Bryant that "the nature of the cicatrix varies with the tissue in which it is formed, the new connecting medium or cicatrix under all circumstances having a powerful tendency to adapt itself to the peculiar character of the tissue in which it is placed. The consolidating, reperative material in every instance partaking of the character of the parts with which it is connected."

